# Case Report: Pars plana filtration in the treatment of nanophthalmos patients with secondary angle closure glaucoma

**DOI:** 10.3389/fmed.2026.1741824

**Published:** 2026-04-13

**Authors:** Chang Jiang, Li Tang, Xiaobin Xie, Guofan Cao, Xiumiao Li

**Affiliations:** 1 The Affiliated Eye Hospital of Nanjing Medical University, Nanjing, China; 2 The Fourth School of Clinical Medicine, Nanjing Medical University, Nanjing, China; 3 Department of Ophthalmology, Beijing Shijitan Hospital, Capital Medical University, Beijing, China; 4 Eye Hospital, China Academy of Chinese Medical Sciences, Beijing, China

**Keywords:** angle-closure glaucoma, case report, malignant glaucoma, nanophthalmos, pars plana filtration

## Abstract

Nanophthalmos is a rare developmental ocular disorder of congenital origin, manifesting as a notably reduced globe volume, shortened axial length, shallow anterior chamber, thickened sclera, and significant hyperopia. These anatomical features predispose affected individuals to angle-closure glaucoma as a result of anterior segment crowding. Traditional treatments for nanophthalmos-related angle-closure glaucoma often involve complex combined surgeries, which are not only technically demanding but also easily cause postoperative complications, such as malignant glaucoma and choroidal leakage. This case series presents three middle-aged and elderly female patients (a total of four eyes) with nanophthalmos-related angle-closure glaucoma. Common clinical findings included an axial length ≤ 20.5 mm, high intraocular pressure (IOP > 21 mmHg; 1 mmHg = 0.133 kPa), shallow anterior chamber, and diminished visual acuity. Based on the pathogenesis of postoperative malignant glaucoma, we implemented a modified surgical technique termed pars plana filtration (PPF). This procedure involves partial excision of the ciliary body to establish an alternative aqueous drainage route, thereby reducing anterior vitreous and posterior segment pressure. Compared to traditional combined surgeries, the PPF approach offers technical simplicity, minimized tissue invasiveness, and a shorter learning curve for ophthalmic surgeons. Our study describes successful implementation of this surgery without postoperative complications observed. These findings suggest that PPF may represent a safe and effective surgical alternative for managing nanophthalmos-related angle-closure glaucoma. Nevertheless, further larger-scale studies are needed to validate its long-term effectiveness and generalizability.

## 1 Introduction

Nanophthalmos is a rare congenital ocular developmental disorder, with a prevalence of approximately 1.0%–1.5% of individuals (1, 2). It is characterized by a shortened axis length, shallow anterior chamber, thickened sclera, and significant hyperopia, typically presenting bilaterally and symmetrically (3, 4). The reduced eyeball volume–approximately two-thirds of the normal size–accompanied by a normally sized lens leads to crowding of the anterior segment (5). This anatomical configuration predisposes patients to refractory angle-closure glaucoma (1, 6, 7). Conventional glaucoma surgeries often yield suboptimal outcomes in these cases due to the high risk of complications such as malignant glaucoma, choroidal leakage, and retinal detachment (6, 8). Therefore, complex combined surgical approaches are typically employed, such as trabeculectomy combined with sclerectomy (9–11), punch sclerostomy (12), and cataract phacoemulsification combined with vitrectomy (9, 13, 14).

To enhance surgical efficacy in nanophthalmos-associated angle-closure glaucoma, we designed a novel technique termed pars plana filtration (PPF). This procedure creates an independent aqueous drainage pathway, thereby facilitating controlling intraocular pressure and reducing postoperative complications. In this report, we present three patients (four eyes) with nanophthalmos-associated angle-closure glaucoma who underwent PPF. Although this technique has shown promising outcomes, it has not been widely documented in regional or international literature. Our findings aim to provide valuable insights for clinical practice, suggesting that PPF may represent a safer and more effective therapeutic alternative for managing this challenging condition.

## 2 Case presentation

All patients in this case report signed an informed consent form. This case series was reported in accordance with the CARE (CAse REport) guidelines.

**FIGURE 1:**
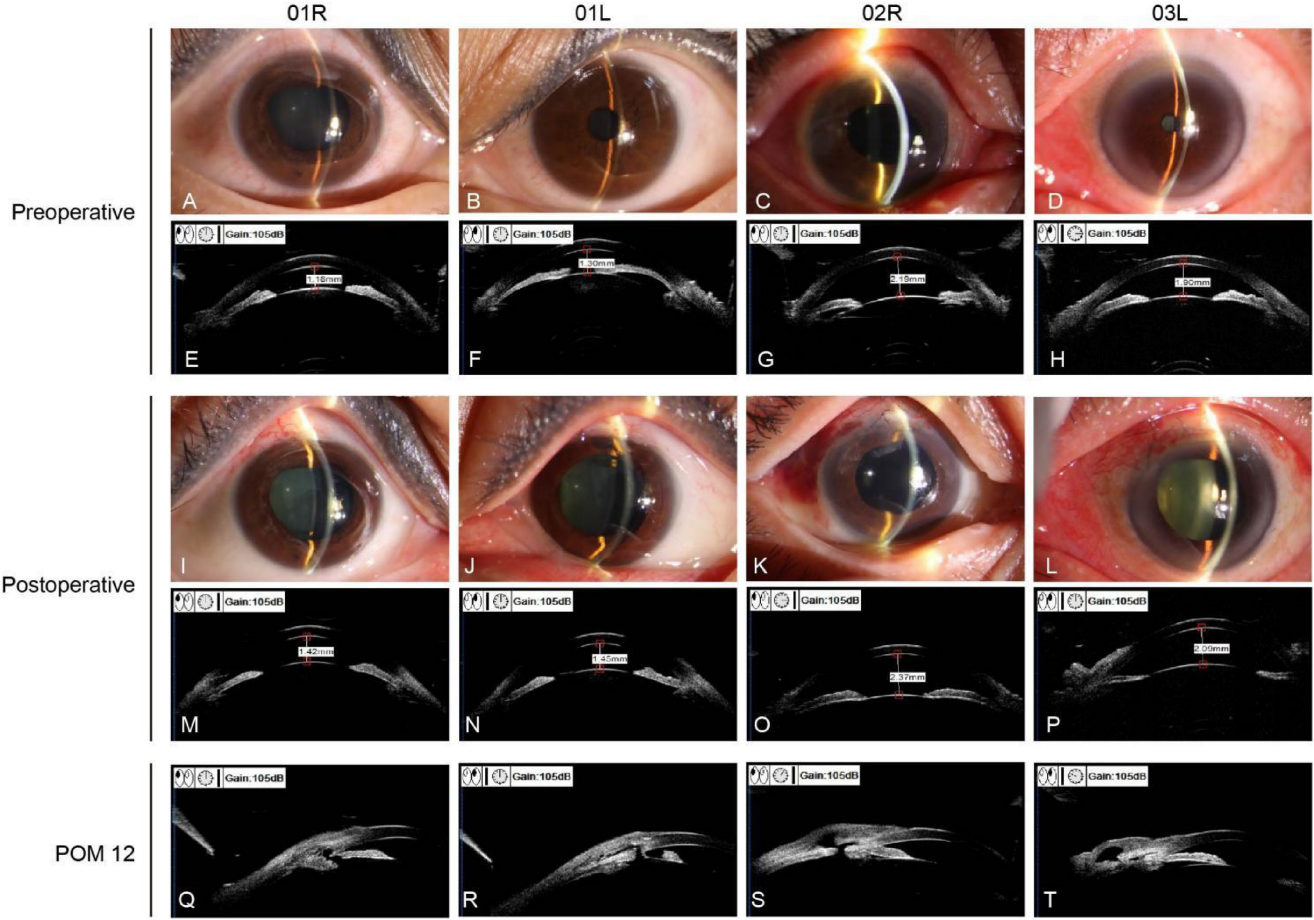
Preoperative and postoperative images of three cases (4 eyes). **(A–H)** Preoperative slit-lamp photographs and ultrasound biomicroscopy (UBM) of all four eyes demonstrated shallow anterior chambers with appositional or synechial angle closure. **(I–P)** Postoperative slit-lamp photographs and UBM revealed significantly deepened anterior chambers with restored anterior chamber depth compared to baseline measurements. **(Q–T)** UBM at 12-months follow-up demonstrated patent filtration blebs with visible fluid-filled subconjunctival spaces and maintained communication with the anterior chamber, confirming long-term patency of the filtration pathway.

Three middle-aged and elderly women (comprising four eyes) were diagnosed with nanophthalmos-associated angle-closure glaucoma. Eligible patients were required to have an axial eye length of ≤20.5 mm (1, 6, 7, 15), persistently elevated IOP in affected eyes exceeding 21 mmHg, a shallow anterior chamber, narrow anterior chamber angle, and decreased visual acuity. All patients underwent comprehensive ophthalmic evaluations, including visual acuity (VA), IOP, slit-lamp examination, ultrasound biomicroscopy (UBM), and B-scan ultrasonography. Axial length (AL), anterior chamber depth (ACD), were measured using the IOL Master 700 (Figures 1A–H and Table 1). Systemic evaluations showed no significant abnormality.

**TABLE 1:** Detailed characteristics of the three patients (four eyes).

Case	Gender	Age (years)	Eye	Surgical method	AL (mm)	IOP (mmHg) (POD -1)	BCVA	ACD (mm)	C/D	Glaucoma diagnosis	B-scan	Surgical history
1	F	40	OD	PPF	17.43	43	0.2	1.18	0.11	AACG	Bilateral VO	None
			OS	PPF	17.31	48	0.25	1.3	0.52			
2	F	49	OD	PPF	16.08	43	0.02	2.19	0.71	AACG	Bilateral VO + PVD	OD: Phaco + IOL + Trab + AV
			OS	–	15.97	15	0.15	1.58	–			
3	F	65	OD	–	19.6	18	LP	–	1	AACG	Bilateral VO	OD: Trab
			OS	PPF	19.39	24	0.2	1.56	0.4			

AL, axial length; IOP, intraocular pressure; BCVA, best-corrected visual acuity; ACD, central anterior chamber depth; VO, vitreous opacity; PVD, posterior vitreous detachment; –, no surgery or no accurate data; Phaco + IOL + Trab + AV, Phaco + IOL implantation + trabeculectomy + anterior vitrectomy.

Case 1: a 40-years-old woman was admitted on May 12, 2024, with complaints of right eye swelling, pain, and vision loss lasting 1 week. Her clinical diagnosis included acute angle-closure glaucoma in both eyes (acute onset in the right eye and preclinical in the left), accompanied by vitreous opacities. Upon admission, she received carteolol hydrochloride, brinzolamide, and brimonidine tartrate eye drops to lower intraocular pressure, as well as levofloxacin eye drops as a prophylactic measure against infection prior to surgery. On the second day after admission, PPF was performed on her right eye under local anesthesia. On the first postoperative day, IOP in the left eye was elevated (R: 26 mmHg; L: 26 mmHg). Despite administering brinzolamide, carteolol hydrochloride, and brimonidine tartrate eye drops to manage IOP, results were suboptimal. On the fourth postoperative day, PPF was performed in the left eye.

Case 2: a 49-years-old woman was admitted on March 17, 2024, reporting diminished vision in the right eye accompanied by swelling and pain for over 1 year, with symptoms worsening in the past 3 months. The clinical diagnosis included post-trabeculectomy acute closed-angle glaucoma and an IOL present in the right eye, acute angle-closure glaucoma (chronic progressive stage) with concurrent cataract in the left eye, uveal leakage syndrome and vitreous opacity in both eyes. Upon admission, she was administered multiple IOP-lowering eye drops to lower IOP, along with levofloxacin for pre-surgical infection prevention. On the second day after admission, PPF was performed on her right eye under local anesthesia.

Case 3: a 65-years-old woman was admitted on June 25, 2023, with complaints of left eye swelling, pain, and vision loss persisting for 2 months. The clinical diagnosis was acute angle-closure glaucoma (left anterior phase), post-trabeculectomy optic nerve atrophy with blindness in the right eye, senile cataract and vitreous opacity in both eyes, and hypertension. Upon admission, she received multiple IOP-lowering eye drops to lower IOP, along with levofloxacin for pre-surgical infection prevention. PPF was performed on her left eye under local anesthesia on the second day after admission.

## 3 Surgical procedure

**FIGURE 2:**
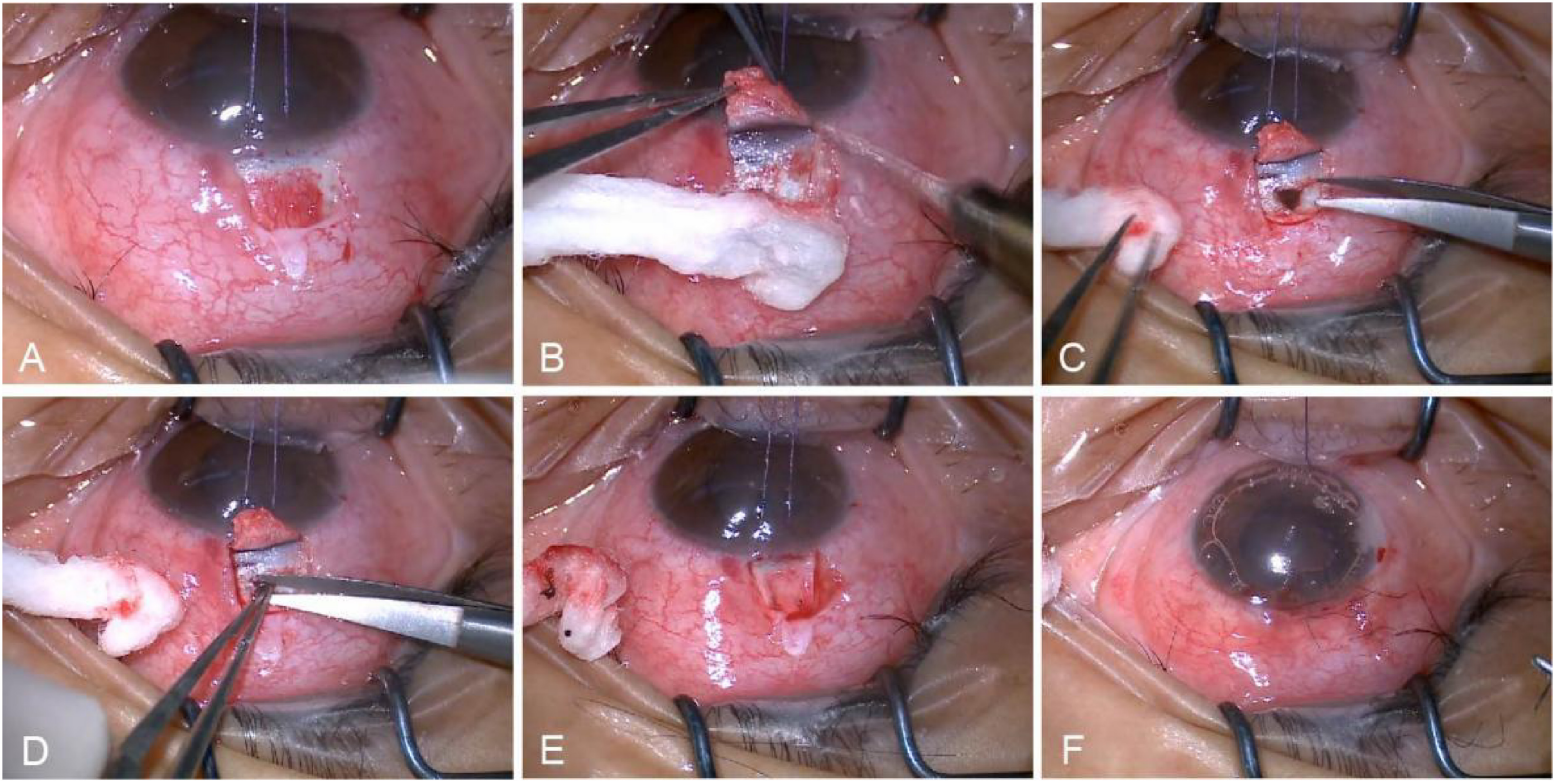
Surgical steps of PPF. **(A)** Make a spherical conjunctival flap with the zone of the fornix as the base. **(B)** Make a lamellar scleral flap based on the limbus. **(C)** Excise deep scleral tissue. **(D)** Excision of the pars plana of the ciliary body. **(E)** Suture scleral flap. **(F)** Suture conjunctival flap.

Standard sterile draping was performed and the operative eye was exposed. Topical anesthesia was achieved by subconjunctival infiltration, and a lid speculum was inserted to maintain globe exposure. A traction suture was placed at the superior limbus to rotate and fixate the eye in downward gaze. A fornix-based conjunctival flap was fashioned and hemostasis was obtained with pinpoint cautery (Figure 2A). A 4 mm × 4 mm limbus-based partial-thickness scleral flap, approximately 50% scleral depth, was then created (Figure 2B). A sponge soaked in 0.02% mitomycin-C was applied beneath both the scleral and conjunctival flaps for 3 min, followed by thorough irrigation with balanced salt solution. Then, at a position 3.5 mm from the corneoscleral limbus, a penetrating resection was performed to remove an approximately 1.5 mm equilateral triangle of deep scleral lamina (Figure 2C), followed by excision of the pars plana of the ciliary body (Figure 2D). This step necessarily disrupts the anterior hyaloid membrane. If vitreous incarceration occurs, it is released; if not, no further manipulation is performed. The scleral flap was re-approximated by anchoring its two free angles with 10-0 nylon sutures (Figure 2E), and the conjunctival flap was repositioned and closed in a watertight fashion (Figure 2F). At the conclusion of the procedure, an antibiotic–corticosteroid ointment was instilled into the conjunctival sac, and the eye was dressed with a sterile pad and shield.

## 4 Results

The three patients reported in this study had an axial length of ≤20.5 mm and were diagnosed with closed-angle glaucoma based on persistent elevated IOP (>21 mmHg), shallow anterior chamber (anterior chamber depth < 2.5 mm in all cases), narrowing of the anterior chamber angle (as confirmed by UBM), and decreased visual acuity (Figures 1A–H and Table 1). We performed pars plana filtration (PPF) surgery on four eyes of these three patients. Of the four eyes involved, one had previously undergone combined surgery comprising “Phaco + IOL implantation + trabeculectomy + anterior vitrectomy” (Table 1). Intraoperatively and postoperatively, no complications occurred in any of the four eyes, including malignant glaucoma, choroidal effusion, or retinal detachment. Comparative analysis of preoperative and postoperative slit-lamp photographs and UBM images demonstrated a significant increase in anterior chamber depth and alleviation of anterior segment crowding (Figures 1I–P and Table 2).

**TABLE 2:** Surgical method, pre-and postoperative central anterior chamber depth (ACD), follow-up of intraocular pressure (IOP), follow-up of best-corrected visual acuity (BCVA) and medication adjustments for three patients (four eyes).

Case		1		2		3	
Eye		OD	OS	OD	OS	OD	OS
Surgical method		PPF	PPF	PPF	–	–	PPF
ACD (mm)	Preoperative	1.18	1.3	2.19	–	–	1.56
	Postoperative	1.53	1.41	2.37	–	–	2.09
IOP (mmHg)	POD -1	43	48	43	15	18	24
	POM 1	24	20	30*	15	32	11
	POM 3	22	23	14	13	45	18
	POM 6	20	22	21	19	37	17
	POM 12	18	24	25*	18	28	15
BCVA	POD -1	0.2	0.25	0.02	0.15	LP	0.2
	POM 1	0.25	0.3	0.08	0.12	NLP	0.3
	POM 3	0.25	0.3	0.08	0.12	NLP	0.3
	POM 6	0.3	0.3	0.08	0.12	NLP	0.25
	POM 12	0.4	0.3	0.08	0.1	NLP	0.25
Drops	Preoperative	PNED + Alphagan + CCH + BRZ		Alphagan + Azarga + Xalacom + Ganfort		Alphagan + BRZ	
	POD 14	ASEG		CTED + Alphagan + Azarga + TRO		TRO	
	POM 1	ASEG		Alphagan + Azarga + Xalatan		None	
	POM 5	ASEG + TRO + Azarga		Alphagan + Azarga + Xalatan		Xalacom	
	POM 12	TRO + Azarga + Xalatan		Alphagan + Azarga + Xalatan		Xalacom	

–, no surgery or no accurate data; *, self-discontinuation of latanoprost eye drops; PNED, pilocarpine nitrate eye drops; Alphagan, brimonidine tartrate eye drops; CCH, carteolol hydrochloride eye drops; BRZ, brinzolamide; Azarga, brinzolamide and timolol maleate eye drops; Xalacom, latanoprost/timolol maleate eye drops; Ganfort, bimatoprost and timolol maleate eye drops; ASEG, atropine sulfate eye gel; TRO, tropicamide; CTED, compound tropicamide eye drops; Xalatan, latanoprost eye drops.

Surgical outcomes were evaluated according to standardized criteria. Following the World Glaucoma Association (WGA) and European Glaucoma Society (EGS) guidelines for reporting glaucoma surgical trials, and considering the success criteria established by Fan et al. (9, 16) for complex nanophthalmos cases, surgical success was defined as the simultaneous fulfillment of all three criteria: (1) IOP control between ≥5 mmHg and ≤21 mmHg, or reduction > 30% from baseline; (2) absence of severe complications including malignant glaucoma, suprachoroidal hemorrhage, retinal detachment, endophthalmitis, or loss of light perception; (3) stability without need for additional glaucoma surgery during follow-up. Complete success required meeting all criteria without medications, whereas qualified success permitted adjunctive IOP-lowering medications.

At 6 months postoperatively, no eyes (0/4, 0%) achieved complete success, whereas all eyes (4/4, 100%) achieved qualified success. The mean IOP reduction was 47.0% ± 11.0%, from a preoperative mean of 39.5 ± 12.4 mmHg to 20.0 ± 2.2 mmHg at 6 months. Concurrent with these IOP outcomes, visual acuity and best corrected visual acuity (BCVA) improved in all patients compared with preoperative values. Because the preoperative IOP exceeded 40 mmHg in three of four eyes, accurate visual field testing and visual function assessment could not be performed. Therefore, only visual acuity comparisons are presented in the follow-up table (Table 2).

Before surgery, three patients were on multiple topical antiglaucoma medications, yet IOP remained completely uncontrolled and persistently high. Postoperatively, IOP was basically maintained within the normal range with adjunctive medication (Table 2). During the postoperative follow-up period, IOP-lowering medications were used as adjunctive therapy to achieve individualized target pressures aimed at preventing further optic nerve damage. No bleb needling was performed on any of the four eyes, and UBM at the 1-year follow-up demonstrated patent filtering blebs (Figures 1Q–T). All four eyes completed at least 1 year of postoperative follow-up. During this period, IOP and visual acuity remained stable in all cases (Table 2).

## 5 Discussion

Following the development of the primary optic vesicle, arrested ocular growth results in congenital microphthalmia (17). When both the anterior and posterior segments are proportionally underdeveloped, the condition is termed nanophthalmos (6, 18). Clinical features of nanophthalmos typically comprise shortening of both the anterior and posterior ocular axes, shallowing of the anterior chamber, scleral thickening, and significant hyperopia (3, 4). The unique ocular structure in nanophthalmos significantly increases the risk of angle-closure glaucoma (1, 19), with reported incidence rates ranging from 35.7% to 77%. This condition most commonly manifests between the ages of 40 and 60 (20), frequently presenting with a narrow anterior chamber angle and elevated intraocular pressure upon diagnosis. Notably, patients with nanophthalmos-associated closed-angle glaucoma are particularly susceptible to malignant glaucoma following glaucoma surgery. This susceptibility is primarily attributable to the crowded anterior segment, oversized lens, and thickened scleral tissue.

When maximal tolerated medical therapy combined with laser iridotomy/iridoplasty fails to control IOP in patients with nanophthalmos-related secondary angle-closure glaucoma, conventional filtration surgery is typically considered as the subsequent intervention, despite its association with frequent intra- and postoperative complications (6). To reduce surgical risks, modifications to the standard technique are often adopted, either through alternative filtration approaches or by combining filtration surgery with adjunctive procedures, such as sclerectomy (9–11), punch sclerostomy (12), vortex decompression (11), lensectomy and IOL implantation (9, 13, 21, 22). While combined sclerectomy and vortex vein decompression may lower scleral wall tension, and punch sclerostomy can facilitate drainage of choroidal effusion, certain nanophthalmic eyes remain susceptible to postoperative uveal effusion and malignant glaucoma (6, 10–12). A combined approach involving vitrectomy, lensectomy, and IOL implantation has been reported to decrease the incidence of postoperative uveal effusion by reducing intraocular volume, deepening the anterior chamber, and releasing anterior hyaloid adhesions (6, 17, 21, 23). Nevertheless, the simultaneous removal of the lens and vitreous body entails considerable ocular trauma and potential structural compromise.

In response to the distinctive anatomical features of nanophthalmos and the heightened risk of postoperative malignant glaucoma, we developed pars plana filtration (PPF), a surgical technique that has demonstrated favorable outcomes. Nanophthalmos is marked by thickened sclera and a shallow anterior chamber, leading to a self-perpetuating cycle in which elevated posterior segment pressure intensifies anterior crowding and angle closure. The underlying pathophysiology includes compromised trans-scleral fluid outflow and possible vortex vein compression, contributing to venous congestion and fluid accumulation in the posterior segment. Conventional trabeculectomy in these cases may further reduce anterior chamber depth, displace the iris-lens diaphragm anteriorly, and trigger malignant glaucoma. In contrast, PPF establishes an independent drainage pathway via the pars plana region, effectively lowering pressure in the anterior vitreous and posterior segment, which stabilizes the iris-lens diaphragm against forward movement. By doing so, PPF relieves anterior segment crowding and corrects aqueous misdirection while preserving the native anatomy of the anterior chamber. This approach directly interrupts the pathological cycle of posterior pressure driving anterior crowding specific to nanophthalmos, thereby significantly lowering the incidence of refractory shallow anterior chamber and alleviating ciliary block (24).

Current surgical options for nanophthalmos primarily consist of combined pars plana vitrectomy (PPV) and phacoemulsification, procedures that are associated with considerable trauma and elevated rates of postoperative complications (e.g., uveal effusion, choroidal hemorrhage, corneal decompensation). Similarly, conventional trabeculectomy and anterior chamber tube shunts (e.g., Ahmed or Baerveldt implants) may aggravate anterior crowding or precipitate malignant glaucoma as a consequence of intraoperative manipulation within the crowded anterior chamber. In contrast, PPF achieves intraocular pressure reduction through a less invasive technique that preserves lens integrity and ocular anatomy. This approach helps maintain preoperative visual function, improves surgical safety, and lowers the incidence of postoperative complications, including hypotony and infection. Relative to multi-step combined surgeries, PPF offers notable benefits in terms of procedural simplicity, reduced tissue trauma, and a shorter learning curve. A key distinguishing characteristic of PPF is its physiological mechanism of posterior segment decompression, which directly addresses the underlying pressure imbalance in nanophthalmic eyes while respecting the native anterior chamber architecture. Importantly, these advantages are attained without sacrificing therapeutic effectiveness, as demonstrated by durable intraocular pressure control and anatomical restoration in our clinical series.

This study yielded informative outcomes but has several limitations. First, all 4 eyes exhibited media opacities that precluded clear fundus visualization, and miotic pupils were observed in Cases 1 and 3 (Figures 1A–D). Given dangerously elevated IOP (>40 mmHg), pupillary dilation was contraindicated. Consequently, reliable preoperative fundus photography, CDR assessment, and visual field testing were unattainable. Among the four eyes, two presented with VA < 0.1, rendering perimetry meaningless; in the remaining eyes, markedly elevated IOP and associated macular/retinal edema further compromised the accuracy of both visual field testing and OCT imaging. Moreover, the retrospective nature, rarity of the condition, limited sample size, and absence of a control group may have introduced confounding factors. Although our 1-year data indicate that PPF is safe and yields stable outcomes, extended monitoring and larger-scale investigations are warranted to establish broader applicability and long-term performance.

This novel approach offers a minimally invasive, safe, and effective treatment alternative that may substantially alleviate both the economic and psychological burden on patients. Although not encountered in our series, potential risks including hypotony maculopathy, prolonged choroidal effusion, or delayed-onset infection warrant consideration in future cohorts. PPF as a standalone procedure may be insufficient for advanced cases requiring very low target IOPs without medication. For patients with nanophthalmos-related secondary glaucoma who have absolute surgical indications–specifically visually significant eyes with medically refractory IOP–the choice of technique should be individualized based on specific ocular characteristics.

## Data Availability

The original contributions presented in this study are included in the article/supplementary material, further inquiries can be directed to the corresponding authors.
